# Modeling enzyme-ligand binding in drug discovery

**DOI:** 10.1186/s13321-015-0096-0

**Published:** 2015-10-06

**Authors:** Janez Konc, Samo Lešnik, Dušanka Janežič

**Affiliations:** National Institute of Chemistry, Hajdrihova 19, SI-1000 Ljubljana, Slovenia; Faculty of Mathematics, Natural Sciences and Information Technologies, University of Primorska, Glagoljaška 8, SI-6000 Koper, Slovenia

**Keywords:** Unknown protein fuctions, Off-target binding, Drug repositioning, Ligand 3D homolgy modeling, Induced-fit simulations, ProBiS-ligands web server

## Abstract

Enzymes are one of the most important groups of drug targets, and identifying possible ligand-enzyme interactions is of major importance in many drug discovery processes. Novel computational methods have been developed that can apply the information from the increasing number of resolved and available ligand-enzyme complexes to model new unknown interactions and therefore contribute to answer open questions in the field of drug discovery like the identification of unknown protein functions, off-target binding, ligand 3D homology modeling and induced-fit simulations.

## Background

**Fig. 1:**
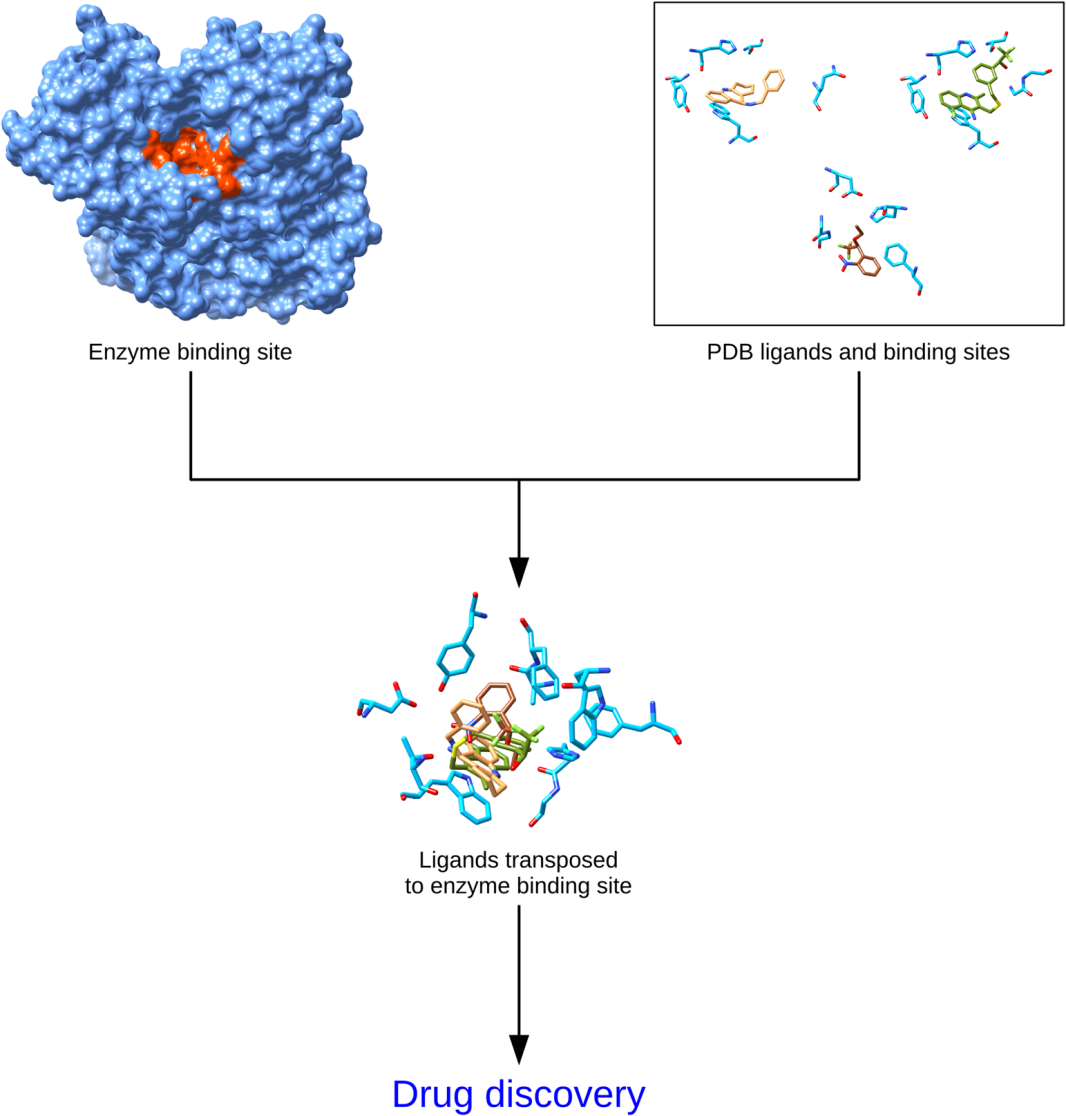
Flowchart of binding site comparison with subsequent ligand transposition

Predicting ligands that bind with sufficient strength to a corresponding protein is a challenging task in biochemistry and has significant implication in the discovery of new drug candidates. Many approaches have been developed for this task; the most commonly used being molecular docking [1]. However one of the main drawbacks of classical template-free docking is that every molecule is docked ab initio, and no information from existing similar protein–ligand complexes is taken into consideration. Therefore alternative approaches that use information from existing protein–ligand complexes, which can be obtained from freely-available databases, such as the Protein Data Bank [2] are becoming increasingly important. The main assumption of such approaches is that similar protein binding sites bind similar ligands, and thus a known ligand from one protein can be transposed to a similar binding site in another protein that was previously not known to bind this ligand. Transposition of ligands is based on accurate alignments of three-dimensional amino-acid patterns or of their corresponding functional groups in the proteins’ binding sites; due to their local nature in the binding sites, such alignments may not be possible with standard sequence or structure alignment approaches. Ligand transposition shown in Fig. 1 can thus be a powerful approach, which can be used in pharmaceutical applications such as drug repositioning [3–6], ligand-homology modeling [7–9], induced-fit simulation [10] and binding site prediction [11–13]. Information about the software described in the following sections is available in Table 1.

**Table 1:** Software packages for modeling protein–ligand binding using ligand transposition

Name	URL	Description	Availability
ProBiS	http://www.probis.cmm.ki.si/	Detects structurally similar binding sites without reference to known binding sites	Freely-accessible web server
GalaxySite	http://www.galaxy.seoklab.org/site/	Combines binding site information from known proteins with molecular docking to predict ligand binding amino acid residues	Freely-accessible web server
Surflex-PSIM	http://www.biopharmics.com/downloads.html	Fully automated ligand binding pocket detection and comparison based on surface similarities to other known proteins	Not freely-accessible
POP	https://www.sites.google.com/site/offtargetpipeline/	Integrated computational method for proteome-wide off target identification	Free for academic users and not-for-profit institutions
FINDSITE^comb^	http://www.cssb.biology.gatech.edu/FINDSITE-COMB/	Threading/structure-based, proteomic-scale virtual ligand screening approach	Freely-accessible for web server for academic users
ProBiS-ligands	http://www.probis.cmm.ki.si/ligands/	Detects and transposes ligands between similar binding sites	Freely-accessible web server
FragFEATURE	https://simtk.org/home/frag-feature/	A machine learning approach to predict small molecules fragments preferred by a target	Freely-accessible

## Function prediction

Proteins interact with one another and with other molecules, mediate metabolic and signaling pathways and thus regulate cellular processes [14]. One of the fundamental tasks of proteins is to act as enzymes [15], i.e., biological catalysts that increase the rate of practically all chemical reactions that are taking place within cells. Due to their central role in biological function, they control mechanisms leading to healthy and diseased states in the organism. However, for a substantial number of proteins, and subsequently enzymes, their functions are not known, therefore an important challenge in structural genomics is the prediction of function of these uncharacterized proteins [16]. While experimental determination of a protein function is still the most reliable way to characterize unknown proteins, it is difficult to prioritize functional experiments among the many possible functions a protein could perform. To guide experimentalist, a number of computer approaches are routinely used to predict protein function. However, many are based on sequence and overall structural homology [17, 18], consequently often missing similarities when only local binding sites are conserved. The identification of such binding sites on the protein surface is therefore usually the starting point for protein function annotation. Moreover, because knowledge of the binding site location is the prerequisite for molecular docking, binding site identification is often a first step in structure-based drug design.

Based on the idea that proteins with similar local binding sites perform similar function, an algorithm ProBiS and its corresponding web server [19, 20] was developed. ProBiS uses a fast maximum clique algorithm [21] to compare a query protein to members of a database of 3D protein structures and detects with sub-residue precision structurally similar binding sites as patterns of physiochemical properties on the protein surface. The algorithm thus identifies database proteins that share local structural similarities with the query protein independent of the global protein folds, and generates structure-based alignments for every query protein-database protein pair. Moreover, structural similarity scores are calculated for the query protein surface residues, and are expressed as different colors on the protein’s surface. ProBiS was tested for its binding site detection ability on a set of 39 protein structures and by comparing it to an evolutionary conservation mapping method ConSurf [22] and an energy-based method Q-SiteFinder [23], it was shown that ProBiS outperformed both of the two aforementioned methods. Moreover, to demonstrate ProBiS’ unique ability to detect and align similar binding sites in the absence of global fold similarity, the authors examined 10 pairs of protein structures, where the two members of each pair exhibited different folds but had known similar binding sites and performed a similar function. ProBiS was compared to three different structural alignment algorithms; DaliLite [24], MolLoc [25] and MultiBind [26]. The comparison between the methods was made by calculating the RMSD between previously identified, similar binding site residues, after the proteins in the pair have been superimposed. ProBiS demonstrated, by far, the lowest average RMSD in comparison to other methods; while also having the ability to align binding sites in an unsupervised fashion, which allows it to perform automatic, large database searches. ProBiS, in combination with molecular dynamics, was also used to propose the function to a protein of unknown activity—the Tm1631 protein from *T. maritima* [27]. The binding site comparison revealed numerous similarities with nucleotide binding sites; including specifically, a DNA-binding site of endonuclease IV. Based on the superimposition of Tm1631 with endonuclease IV, a hypothetical model of the Tm1631-DNA complex was constructed. This model was validated with the use of CHARMM [28, 29] to perform a molecular dynamics simulation, which showed that the interactions predicted by the Tm1631-DNA model correspond well to those known to be importation in the endonuclease IV-DNA complex. The simulation also showed that the binding free energies of the model and the known complex were in close agreement. The authors thus concluded that the Tm1631 protein could be a DNA binding enzyme with endonuclease activity. ProBiS was also used to identify conserved binding sites on hemagglutinin, a protein responsible for binding the influenza virus to cells [30]. A local structural superimposition across all subtypes and strains of hemagglutinin available in the PDB at the time, revealed a new conserved region on hemagglutinin, a potential conserved target for influenza drug and vaccine development.

Another freely-accessible web server for binding site annotation—GalaxySite [31] combines binding site information from known proteins with molecular docking to predict ligand binding amino acid residues. Initially the server uses HHsearch [32] to search for similar protein–ligand complexes in the structural databases. The highest ranking ligands are transposed from the target to the query structure where their binding conformation is optimized using the LigDockCSA protein–ligand docking program [33]. Binding-site residues are then identified based on their proximity to the docked ligand. GalaxySite was extensively tested on different ligand binding prediction test sets, on which it showed superior or similar performance compared to other state of the art prediction methods.

Moreover, Surflex-PSIM [34], a novel method combining initial binding site recognition with subsequent binding site comparisons has been developed. The newest version of Surflex-PSIM is able to automatically detect ligand binding pockets and compare them, based on their surface similarity to other binding sites extracted from large protein databases (e.g. PDB). The method was tested on a set of eight proteins, whose function was unknown at the time of the testing. All of the eight proteins were screened against ~60,000 ligand binding sites from the PDB. Surflex-PSIM correctly identified functional matches that predated query protein biochemical annotation for five out of the eight proteins. In addition, 12 currently unannotated proteins were also screened, resulting in a large number of statistically significant binding site matches, which could suggest likely functions for these uncharacterized proteins. Surflex-PSIM was also used as a part of a combined computational approach which identified known PPARα agonists as also being cyclooxygenase (COX) inhibitors [35]. Pockets of 9 COX enzymes were compared to 14 human PPARα binding pockets and the method identified high similarity of pocket surfaces between proteins with the PDB codes 2rew (PPARα receptor) and 3rr3 (COX-2 enzyme). Subsequent experiments confirmed that fenofibric acid, a known PPARα agonist, does in fact inhibit, in a does dependent manner, both the COX-1 and COX-2 enzymes.

## Drug repositioning

Drug repositioning or repurposing is a principle of discovering novel therapeutic indications for existing approved drugs, which provides an alternative and cost-efficient strategy of discovering disease therapeutics [36]. A prerequisite for drug repurposing is drug promiscuity (polypharmacology), which is a drug’s ability to bind to several different targets. A recent study suggests that the most important factor contributing to the observed promiscuity of many drugs is the local binding site similarities between different protein targets [3]. It was discovered that off-target binding is the major cause of unwanted side-effects for many drugs from a wide range of therapeutic areas [37]. Therefore binding site comparison methods may have an important role in identifying the polypharmacological activity of molecules.

Recently, an integrated computational method for proteome-wide off target identification, abbreviated POP (proteome-wide off-target pipeline) was developed. POP combines ligand binding site comparison analysis, protein–ligand docking and electrostatic potential calculation to identify possible promiscuous protein–ligand interactions throughout the proteasome. The core component of this method is the well-established software for binding site comparison SMAP [38, 39]. SMAP initially detects the location and boundary of the query protein ligand binding site. The binding site is then compared against target 3D protein structures using a sequence-order independent profile–profile alignment (SOIPPA) algorithm [39] that is able to detect similar binding sites between structurally unrelated proteins. The next step is the superimposition (and subsequent ligand transposition) of the query binding site to the top scoring target binding sites. The transposed ligand binding pose serves as the starting structure in the following docking and scoring. The highest scoring protein–ligand complexes reflect the possible off-targets of the ligand. In addition, the electrostatic potential binding energy and similarity between the binding sites can be calculated based on the binding pose of the ligand. POP was applied to identify possible off-targets of the HIV protease inhibitor nelfinavir. The protein–ligand complex of HIV protease bound with nelfinavir (PDB code: 1ohr) was searched against a variety of human protein structures and models. Top ranked hits contained multiple members of the protein kinase superfamily, most of which are on the upstream of the Akt1 and Akt2 enzymes in the AKT pathway [40], which is in correlation with the experimentally observed anti-cancer effect of nelfinavir.

## Ligand 3D homology modeling

Virtual screening is a widely used approach in the early stage of pharmaceutical discovery [41]. In practice, we distinguish two broad categories of virtual screenings: (a) ligand based and (b) structure based [42, 43]. Ligand based virtual screening is relatively fast, however the method requires previously known ligands that bind to a certain target; this hinders its universality and large-scale application. On the other hand, structure based virtual screening uses the structure of a target/target binding site, to which it docks potential drug molecules and evaluates the binding likelihood using different scoring functions; its main advantage is that no prior knowledge of known active ligands is needed. The main disadvantage of structure based virtual screening is the requirement for high-resolution structures of target proteins, which are in many cases not available, especially for G-protein coupled receptors and ion-channels [44]. However, with increasing number of holo protein structures deposited in large protein databases, a novel type of virtual screening, termed ligand-homology modeling (LHM) is gaining recognition [7–9, 45]. LHM is a knowledge-based approach that relies on the fact that evolutionary related proteins share similar functions and thus bind similar ligands, and that this information can be used to predict ligand-target interactions. In general, LHM algorithms start with identifying and aligning similar binding sites between a query and target database holo proteins, which is then followed by transposing the ligands from target proteins to the query binding site.

One of the main shortcomings of the LHM is the need for sufficient numbers of holo proteins that share similar binding sites to the query protein. To overcome this serious disadvantage, a novel combined ligand homology modeling approach, FINDSITE^comb^ was developed [46, 47]. FINDSITE^comb^ is a composite approach consisting of the FINDSITE^filt^ and the FINSDSITE^X^ algorithms. While the former still uses known holo proteins deposited in protein databases, FINSDSITE^X^ circumvents this requirement by employing homology modeling to model structures of target proteins not yet available in protein databases. The FINDSITE^filt^ algorithm works by initially finding template holo protein structures that are evolutionary related to the target structure from the PDB database. Next, a heuristic structure–pocket alignment method is used to superimpose template pockets to the target structure and a sequence dependent scoring function to rank the best templates (based on the structure-pocket alignment) from whose their corresponding ligands will be used as reference small molecules in subsequent ligand based virtual screening. FINDSITE^X^ on the other hand, as mentioned, employs homology modeling methods to model structures of template proteins, which are then aligned and compared to target proteins using a global structure alignment method. Similar as in FINDSITE^filt^, the ligands of the top ranked templates are used as reference small molecules for ligand based virtual screening, where the appropriate template ligands are obtained from the DrugBank [48] and ChEMBL [49] databases. By combining the scores obtained from FINDSITE^filt^ and FINDSITE^X^ ligand based virtual screening runs, the method evaluates the likelihood of each compound of being a true active. The performance of FINDSITE^comb^ was thoroughly evaluated both on the directory of useful decoys (DUD) database [50] and experimentally on eight different protein targets [51]. In the former evaluation, FINDSITE^comb^ outperformed, in most cases, established docking methods, such as AUTODOCK Vina [52] and DOCK 6 [53]; demonstrating significantly higher enrichment factors and areas under the ROC curves. Testing FINDSITE^comb^ on eight different proteins with subsequent experimental ligand binding evaluations, the authors identified 47, mostly novel small molecules with µM or better affinities. Of those, 10 ligands showed affinities in the nanomolar range (for dihydropholate reductase from E.*coli* and two mammalian protein tyrosine phosphatases).

**Fig. 2:**
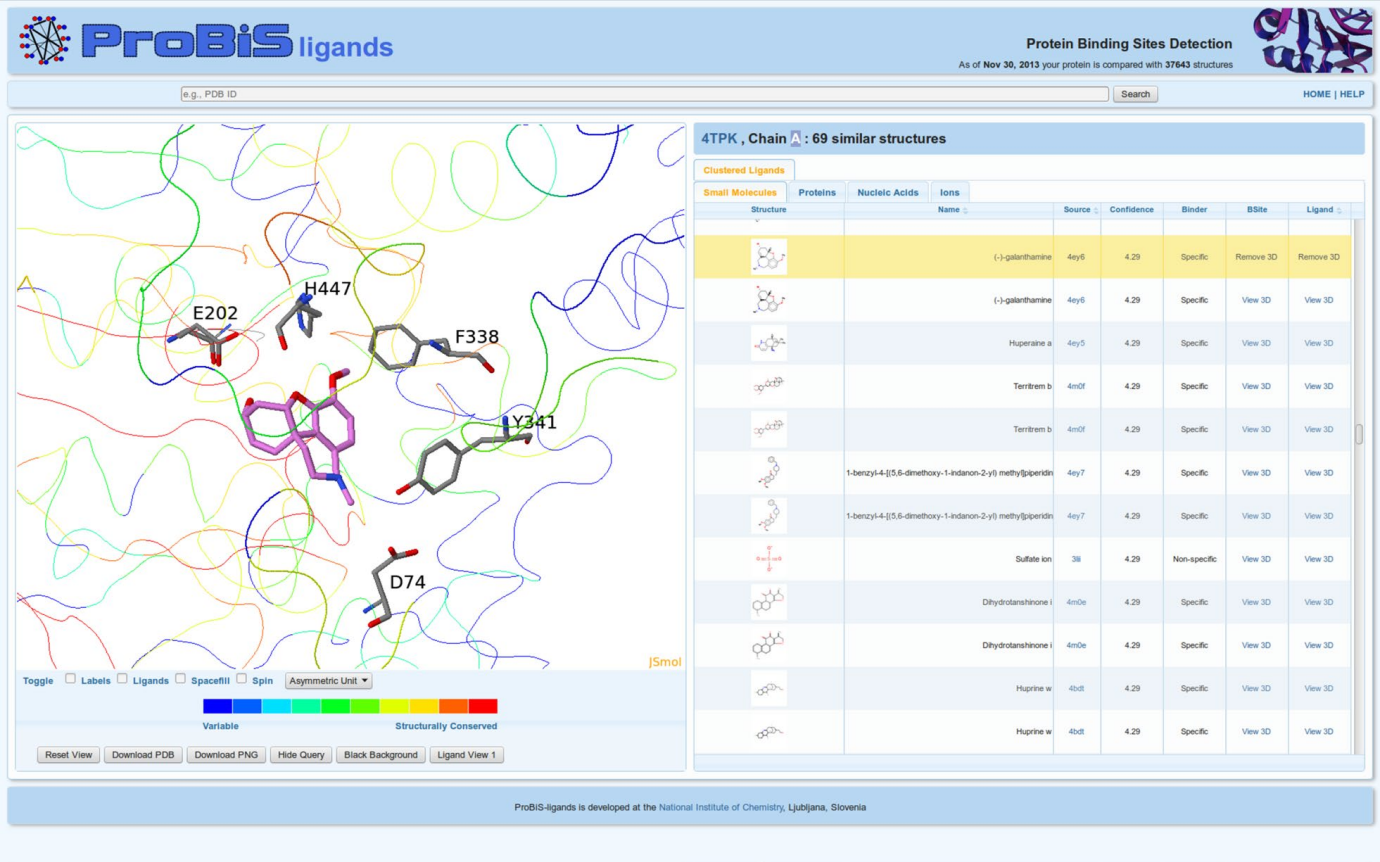
Ligand homology modeling using ProBiS-ligands web server on the example of butyrylcholinesterase enzyme (PDB code: 4tpk). On the *right side* of the screen is the list of predicted ligands with their corresponding Z-scores, specificities and PDB codes of protein structures from which they were transposed. The selected ligand’s *row* is highlighted *orange*. On the *left side* is the Jsmol viewer that contains the three-dimensional pose of the selected predicted ligand (galantamine, sticks, *violet*) and the predicted binding amino-acid residues (sticks, CPK colors, *black labels*)

Also a recently developed, freely-available web server, which enables ligand homology modeling, is ProBiS-ligands (Fig. 2) [45], a method that uses a fast maximum clique algorithm [21] to screen the non-redundant PDB database, to find structurally similar target binding sites to user provided input query protein, independent of their sequence or global structural similarity. Ligands which are bound in the identified similar binding sites are transposed to the query protein by rotation and translation of their atoms’ coordinates generated by the superimposition matrices acquired from the initial superposition of the query and target proteins. ProBiS-ligands is able to predict protein–protein, protein-small molecules, protein-nucleic acid, and protein-ion interactions. The performance of the web server was assessed with a test set [54] containing 500 proteins models and their corresponding experimental structures. The success of ligand prediction was measured by calculating the correspondence between the predicted ligand binding sites, i.e. query residues <4 Å from the first cluster of predicted small molecules or ions and the actual known binding sites for each of the 500 proteins. This binding site prediction results were evaluated with the Matthews correlation coefficient, precision and recall (for definitions of each see, e.g. [54]). Moreover to assess the similarity of predicted ligands with the actual ones, each highest scoring (by Z-score) predicted ligand from the first small molecule or ion cluster was compared with the actual known ligands using an in-house developed 2D molecular graph matching algorithm. Ligand similarities were expressed as the Tanimoto coefficients and were averaged across all comparisons. ProBiS-ligands showed encouraging results, with the average ligand similarity Tanimoto coefficient of 0.61 (0.55) and MCC of 0.54 (0.41) (the values in parenthesis are for the modeled structure). Even when all templates sharing >10 % sequence identity with the target were removed (to simulate the lack of similar templates that frequently occur in real world simulations), reasonable predictions for the experimental protein structures were still possible with the average ligand similarity of 0.40 and MCC of 0.28. In contrary, for protein models, templates of at least 20-30 % sequence identity were required to obtain similar accuracy.

As discussed, an important drawback in classical screening methods is that large molecular databases (usually containing millions of compounds) have to be screened to find potential drug candidates. Given this difficulty of thoroughly exploring the chemical-space of drug like molecules, fragment-based approaches have emerged. Fragments refer to low-molecular-weight molecules, usually 140–300 Daltons in weight [55, 56], that can be connected to form larger molecules. It was shown that fragments have higher hit rates compared to large, complex drug-like molecules because they are less likely to possess suboptimal interactions or physical clashes with the target [57]. Moreover, a fragment library can provide a more compact and tractable basis set for chemical space than standard small molecule libraries [58]. A novel knowledge-based fragment binding predictor FragFEATURE was developed that overcomes many limitations of the existing fragment binding predictors [59]. Using information from the PDB, the authors preliminarily created a database linking local protein structural environments to the small molecule fragments they bind. Given the structural environments from a target protein, FragFEATURE compares them to the database to find similar structural environments and identifies statistically preferred fragments. The results demonstrated the method’s ability to rediscover fragments corresponding to the ligands bound with 74 % precision and 82 % recalls on average. For many protein targets, FragFEATURE was able to identify high scoring fragments that are substructures to known inhibitors. Such predicted fragments can serve as inputs to fragment-based drug design or serve as refinement criteria for creating target-specific compound libraries for experimental or computational screening.

## Induced-fit simulation

One of the main downsides of many established molecular docking algorithms is their inability to consider binding site flexibility [60]. This can present a serious drawback, especially when only a crystal structure without its corresponding ligands (apo structure) is available. Such a protein can be locked in a conformation that is a poor representation of an actual spatial arrangement of binding site amino acid residues upon ligand binding [61], making it a poor candidate for target based virtual screening studies.

To overcome difficulties where the binding pocket conformation is unsuitable for docking, our group developed a new simple and fast approach to simulate the induced-fit conformational changes of protein structures upon binding. For example, the UDP-N-acetylglucosamine enolpyruvyl transferase enzyle enzyme (MurA, PDB code: 1uae) from *E. coli* exists in the PDB only as apoprotein and our initial docking study with the docking program FRED software [62] showed that most drug-like molecules were unable to dock in this binding site without exhibiting severe clashes with the amino acid residues. To circumvent this problem, ProBiS-ligands [45] was used in combination with standard molecular minimization algorithms. With the *E. coli* MurA crystal structural as input, ProBiS-ligands enabled the identification and transposition of a ligand (PDB ligand code: TAV) bound originally in a binding site of the MurA enzyme from *E. cloace* (PDB code: 1ybg). With the new-found ligand transposed to the query binding site and all the original ligands and water molecules removed, molecular minimization was performed on this complex to obtain an open binding site structure of the enzyme that was more suitable for docking. The docking was then repeated on this opened binding site against the ZINC Drugs Now database [63], which yielded very good inhibitors of MurA enzyme as three of the highest scoring compounds that were biochemically tested for their inhibitory ability showed lower than 1 µM IC_50_ values and are now subject for further optimization and research (in preparation).

## Conclusion

Ever increasing numbers of 3D holo enzyme structures deposited in large protein databases enable that the information of known enzyme-ligand interactions be used in predicting and evaluating novel complexes—a key step in drug discovery. Knowledge-based computational methods that apply this information have been successfully used in a wide variety of fields that are of interest to pharmaceutical research; from drug repositioning to simulating induced-fit upon ligand binding. Increased usage of knowledge-based methods for modeling ligand-enzyme interactions is expected in the near future, especially in the early stages of drug discovery processes.
